# Bats seek refuge in cluttered environment when exposed to white and red lights at night

**DOI:** 10.1186/s40462-020-00238-2

**Published:** 2021-01-22

**Authors:** Kévin Barré, Christian Kerbiriou, Ros-Kiri Ing, Yves Bas, Clémentine Azam, Isabelle Le Viol, Kamiel Spoelstra

**Affiliations:** 1 Centre d’Ecologie et des Sciences de la Conservation (CESCO), Muséum national d’Histoire naturelle, Centre National de la Recherche Scientifique, Sorbonne Université, CP 135, 57 rue Cuvier, 75005 Paris, France; 2 Centre d’Ecologie et des Sciences de la Conservation (CESCO), Muséum national d’Histoire naturelle, Station de Biologie Marine, 1 place de la Croix, 29900 Concarneau, France; 3 Institut Langevin, UMR 7587 CNRS, Université Paris Diderot (Paris 7), 1 rue Jussieu, 75238 Paris, France; 4 Centre d’Ecologie Fonctionnelle et Evolutive, UMR 5175, CNRS, 1919 route de Mende, 34293 Montpellier, France; 5 Department of Animal Ecology, Netherlands Institute of Ecology (NIOO-KNAW), PO Box 50, 6700 AB Wageningen, The Netherlands

**Keywords:** Acoustic localization, Artificial light, Flight behaviour, Chiroptera, Microphone array, Streetlight

## Abstract

**Background:**

Artificial light at night is recognized as an increasing threat to biodiversity. However, information on the way highly mobile taxa such as bats spatially respond to light is limited. Following the hypothesis of a behavioural adaptation to the perceived risks of predation, we hypothesised that bats should avoid lit areas by shifting their flight route to less exposed conditions.

**Methods:**

Using 3D acoustic localization at four experimentally illuminated sites, we studied how the distance to streetlights emitting white and red light affected the Probability of bats Flying Inside the Forest (PFIF) versus along the forest edge.

**Results:**

We show that open-, edge-, and narrow-space foraging bats strongly change flight patterns by increasing PFIF when getting closer to white and red streetlights placed in the forest edge. These behavioural changes occurred mainly on the streetlight side where light was directed.

**Conclusions:**

The results show that bats cope with light exposure by actively seeking refuge in cluttered environment, potentially due to involved predation risks. This is a clear indication that bats make use of landscape structures when reacting to light, and shows the potential of vegetation and streetlight orientation in mitigating effects of light. The study nevertheless calls for preserving darkness as the most efficient way.

**Supplementary Information:**

The online version contains supplementary material available at 10.1186/s40462-020-00238-2.

## Background

Artificial light at night (ALAN) is recognized as a prominent and growing threat to global biodiversity [1] and hence there is an urgent need to expand scientific knowledge on its effects on ecosystems, and a demand for efficient solutions to reduce these [2]. ALAN impacts a wide range of taxa, at different spatiotemporal scales [3, 4]. Effects vary from the individual level to the disruption of ecosystem functioning by altering interactions between species and regulatory processes [5–7].

ALAN also affects spatial behaviour by disorienting species and forming barriers in the landscape. For instance, artificial light disorients migrating birds [8] and obstruct toads [9] and highly mobile taxa such as bats [10]. However, the impact of ALAN on species movement across nightscapes remains poorly documented, in particular underlying mechanisms such as changes in spatial behaviour and movement (e.g. flight speed, flight route) of bats in response to light [11] which potentially affects energetic cost and fitness of individuals [12].

This topic is all the more important given that bats are mostly nocturnal and well known to be impacted by ALAN in term of activity [13]. Depending on species, ALAN positively or negatively impacts bat activity (e.g. *Pipistrellus* spp. and *Nyctalus* spp., respectively *Myotis* spp., *Plecotus* spp. and *Rhinolophus* spp.) at the streetlight scale [14–16], while evidence is also accumulating that ALAN negatively impacts activity of these groups at larger scales [17–19].

ALAN also affects bat movement, for example by keeping individuals from crossing of lit gaps in wooded corridors [10] or lit bridges along waterways [20] in urban environments. It was also shown that different spectra reduce commuting activity along hedgerows and that light shy species switch to the unlit side [21].

Among possibilities to reduce these impacts, light spectrum, intensity, directionality, light spill and the duration of lighting are parameters that potentially can be used to reduce negative effects [22]. For instance, light-shy bats such as *Myotis* and *Plecotus* spp. appear to be equally active close to red streetlights and at unlit sites [23]. However, highly light averse species such as *Rhinolophus hipposideros* have shown to avoid all spectra tested for, including red light [21]. Specific part night lighting schemes, with lights turned off from midnight to 5 am, were not found to substantially mitigate effects of light at night as they were still on during the activity peak of bats [14]. Moreover, information on impact distances are also essential to prevent negative effects of lighting setups and allow biodiversity friendly urban planning. However, how the response of bats relates to the distance to light sources – and hence light intensity – is still relatively unknown. Thus far, the only study available on how the response of bats varies with distance reveals clear species dependent differences between 10 and 50 m from a light source [24]. For *Eptesicus serotinus* no difference in effects were shown between 0 and 10 m from the streetlight, however strong negative effects were present between 25 and 50 m from the streetlight [24]. These findings suggest the response of bats to light is intensity dependent.

All these effects of ALAN and possible measures for reduction of impact on bats remain so far mostly studied using activity metrics (i.e. indicators of abundance). However, the level of activity close to a light source does not provide information on how light level affects the behaviour of bats. Indeed, using a single microphone only allows the assessment of average bat activity within an acoustic detection range of approximately five to over 100 m, depending on species specific call amplitude [25].

Therefore, to assess respective effects of light intensity and spectrum on bats, there is a need for the assessment of species-specific changes in flight behaviour (e.g. changes in flight paths). Acoustic localization in three dimensions (3D) is an effective tool for the assessment of flight paths [26]. In a study using this technique, authors found that bats reduce flight height and increase flight speed in presence of artificial light [11]. The attraction of insects by light [27] creates foraging opportunities for bats [28] which should cause bats to reduce flight speed [29]. However, studies found that light increases flight speed, probably due to an increased fear of predation [11, 20]. An alternative solution to reduce predation risk is to avoid open spaces when exposed to light. Hence, we hypothesize that bats that have the opportunity to fly closer to vegetation (i.e. when flying close to the forest edge) seek shelter in the vegetation while getting closer to lights. This hypothesis is supported by the fact that typically highly light-averse species [14, 23, 30] such as *Myotis* and *Plecotus* species, are adapted to fly in cluttered habitats (hereafter named narrow-space foragers) by flying slower and hence more vulnerable to predation by hawking birds. We also hypothesize this behavioural response (i.e. flight closer to vegetation) to begin at least 10 m from the light source as shown by Azam et al. [24]. Indeed, distances from the light source at which behavioural responses (avoidance or attraction) are detected vary according to species, and approximatively lie around 50 m for bat species mostly flying in open space (hereafter named open-space foragers) such as *Eptesicus serotinus*, 10 m for bat species mostly flying at wooded edges (hereafter named edge-space foragers) such as *Pipistrellus* species and at up to 25 m for narrow-space foragers such as *Myotis* and *Plecotus* species [24]. Such distance thresholds correspond to light intensities lower than one lux for narrow-space foragers and between one and five lux for open-space and edge-space foragers [24].

In this study, we hypothesize the distance dependent behavioural response of open, edge and narrow-space foraging bat species to streetlights emitting different spectra. Specifically, in comparison with unlit sites we expect bats to fly closer to the vegetation when getting closer to the light, and as much for spectral composition close to white. Using 3D acoustic localization near experimental light posts in forest edges, we first investigated the probability of bats flying inside the forest versus open habitat in relation with the distance to the light. We studied whether this relationship varies around light posts (e.g. the back- and front side, and above and under the lights) in order to determine the potential of impact reduction by light orientation (i.e. shielding by the light armature).

## Methods

### Experimental sites

**Fig. 1:**
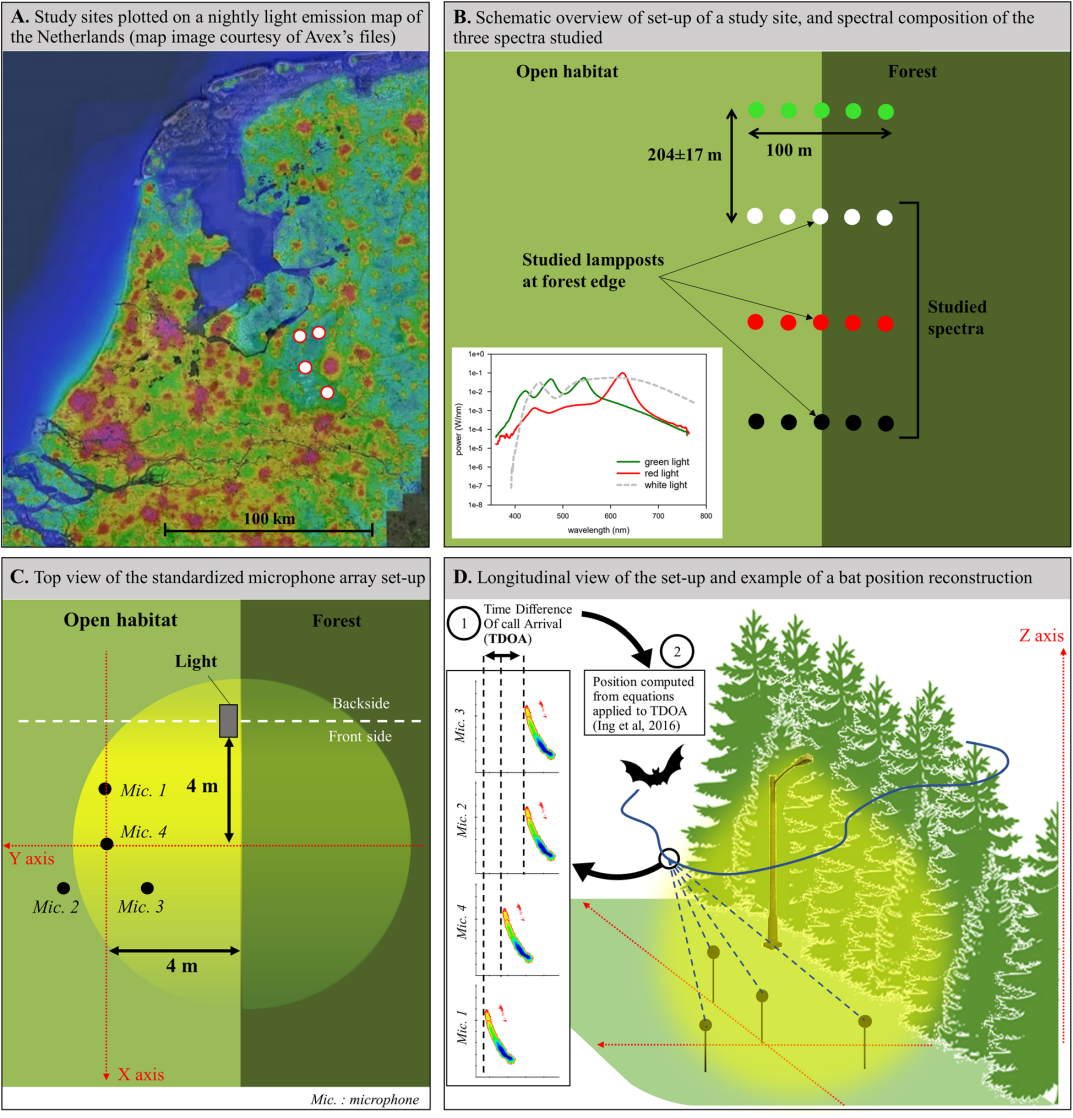
Location of study sites plotted on a nightly light emission map (**a**), schematic overview of set-up of a study site (**b**) and standardized set-up of the microphone array (**c**), and how 3D positions are calculated (**d**). Light posts were always 4 m tall, and always oriented toward the microphone array, parallel to the forest edge

The study was done at four experimentally illuminated sites in The Netherlands, each with four rows (separated by 204±17 m) with five four-meter-tall lampposts (separated by 25 m and the central one at forest edge) placed perpendicular in forest edge habitat (Fig. 1). Each row was randomly assigned to emit white, green or red light (Fortimo white, ClearField red, and ClearSky green light, Philips, Amsterdam, The Netherlands), with one of the rows kept dark (just poles). In this study, we only used the white, red and dark rows. We choose not to study green lights as these have not shown to be an option to reduce impact of light on the activity of bats (and other nocturnally active species) in earlier studies at these sites (Spoelstra et al., 2015, 2017 [23, 31]), and hence to allocate our time and efforts to white and red light. All lights are switched on at sunset, and off at sunrise since spring 2012. All experimental lights emit light in the full spectrum range at low intensity; green lamps have an increased blue and reduced red light emission, and red lamps have an increased red and reduced blue emission (Fig. 1). All light colours have negligible UV emission (see Spoelstra et al. [31] for spectral compositions). The light beam of each light is directed downwards by Philips Residium FGS224 (1xPL-L36WHFP) armatures to project light in preferential directions. The light intensity at ground level is on average 8.7 ± 3.0 lx, which is comparable to the illumination levels of countryside roads (Fig. 1; see Spoelstra et al. [31] for a further description of these experimental sites). For more detail about light intensity in relation with the distance to the lamp and the orientation of the lamp, see Additional file 1, Appendix S1.

### Sampling design and 3D acoustic localization

Bats were acoustically tracked in three dimensions during the first 3 h after sunset for 12 nights between the 10th and 22nd of July 2018. As we could deploy only one microphone array at the time, we unable to simultaneously sample different light treatments, so we sampled white light, red light and dark control during separate nights. In order to limit variation in bat behaviour linked with inter-night environmental variations, we always sampled a different light colour between consecutive nights (Additional file 1: Table S1). All nights were sampled under highly favourable and constant temperatures (average=16.2 °C, Standard Deviation=1.7 °C) and wind speed conditions (average=1.3 m/s, SD=0.8 m/s) (Additional file 1: Table S1). In total four dark control, four red and four white lights were sampled. White light, red light, and dark control were uniquely sampled in two of the four experimental sites, and combinations of the two spectra (dark control and red, and dark control and white, respectively) were sampled in the two other experimental sites. Within the row of light posts, we always sampled the light post right in the forest edge (i.e. at the border of the forest and the open area; see Fig. 1).

To reconstruct 3D positions of bats, we used a trajectography system (hereafter named microphone array) designed at the Institut Langevin (Paris, France). The system uses echolocation calls recorded in a frequency range from eight to 160 kHz at four microphones (FG 3329, Knowles Acoustics, Itasca, IL USA; see Additional file 1, Appendix S2 for more details about sound recording and triggering of echolocation calls). Microphones were arranged in a horizontal triangle form (i.e. three in the corners and one in the middle; Fig. 1). The microphone array was set up similarly near each row: the four microphones were placed in a horizontal plane above the ground surface, with the central microphone in the open space at four meters horizontal distance to the forest edge and to the streetlight axis perpendicular to the forest edge. The microphone array was always placed on the same side of the light (Fig. 1). Bat positions in the detection range of the microphones were continuously assessed using the time of arrival difference (TOAD) of bat echolocation calls between microphones in the array, using the call waveform [26]. Further details about the recording setup, the conversion of bat call arrival times into 3D positions, assigning positions to trajectories and the precision of these can be found in Ing et al. [32] and Additional file 1, Appendices S2 and S3.

### Calculation of the distance from 3D positions to the light and accounting for imprecision

To calculate the distance between each bat position and the light, we used the following equation (*D*, Eq. 1):

1 $$ {D}_i=\sqrt{{\left({x}_i-4\right)}^2+{\left({y}_i-4\right)}^2+{\left({z}_i-4\right)}^2} $$

where *x*, *y* and *z* represent distances to the microphone array for each of the three-dimension axis of a given position *i*. The microphone height was corrected for by entering the actual height of the microphones as placed in the field (i.e. 0.82 to 1 m) in the position calculation software. Since lights were located at the forest edges and were at four meters height, and given the microphone array placement, we subtract four meters to *x*, *y* and *z* axis, in order to compute the real distance to the light (Fig. 1).

Since the imprecision was expected to increase with the distance to the microphone array, we calculated the cumulated imprecision of 3D positions (*I*, Eq. 2) as follows:

2 $$ {I}_i=\sqrt{{\left({dx}_i\right)}^2+{\left({dy}_i\right)}^2+{\left({dz}_i\right)}^2} $$

where *dx*, *yx* and *zx* represent the standard deviation of distances to the microphone array estimated for each of the three-dimension axis of a given position *i* [32]. We choose to discard positions with a cumulated imprecision of more than one meter (Fig. S1) and those not included in any bat individual trajectory (i.e. composed of several positions; for details on trajectory reconstruction see Additional file 1: Appendix S3), which led us to keep 28,646 positions on the 35,067 recorded.

### Assigning species to 3D positions

Individual bat calls used to reconstruct 3D positions were saved by continuously recording sound files. In a second step, sound files were segmented into five-second intervals which is sufficient to cover the average duration of a bat pass [33]. Each of 25,195 five-second files were then classified to the closest taxonomic level using the Tadarida software [34]. Because the identification by echolocation to the species level is difficult, we limited identification to following species groups: the *Eptesicus/Nyctalus* group including *Eptesicus* sp. and *Nyctalus* sp., the *Myotis/Plecotus* group including *Myotis* sp. and *Plecotus* sp., and the *Pipistrellus* group including *Pipistrellus* sp. These three groups respond differently to light: *Eptesicus/Nyctalus* group (i.e. open space forager) are usually considered as light opportunistic or light shy (context dependent), species in the *Myotis/Plecotus* group (i.e. narrow space forager) are light shy, and species in the *Pipistrellus* group (i.e. edge space forager) are light opportunistic.

In a third step, we linked the 3D positions in each 5 s file to the species group found by Tadarida. In case calls of different species groups were found within the same 5 s file, we were able to assign the correct species group to separate series of calls by making use of sequential 3D positions and the peak frequency.

### Statistical analysis

We assessed whether the probability of bats flying inside the forest (PFIF) differed according to the distance to the light, and whether this relationship differed between spectra (i.e. dark control, red and white lights). The relationship between the PFIF and the distance to the light allowed us to define the Flight-Path Switch Distance (FPSD) as the distance at which bats on average flew as much inside as outside the forest. We performed Generalized Linear Mixed Models (GLMM, R package *TMB*), using the PFIF as a binomial response variable where zero corresponded to positions located in the open habitat, and where one corresponded to positions located inside the forest (Fig. 1). We used as explanatory variables the distance to the light, the spectrum type, and the interaction between them to assess the effect of spectra on FPSD. To account for a part of the pseudo-replication (i.e. an average of 15.4 ± 10.1 positions per trajectory; Additional file 1: Fig. S2), we included a random effect on the trajectory identifier. We also included the date as random term in models to control for potential inter-site (i.e. one site sampled each night) and inter-night variations of bat behaviour in relation with lights. Note that weather conditions were highly favourable to bats and stable throughout the sampling period, and that habitat composition was similar between sites (see Sampling design and 3D acoustic localization section and Additional file 1: Table S1).

Given that imprecisions of positions were slightly positively correlated with the distance to the light for *Eptesicus/Nyctalus* group (*r* = 0.05, t = 2.0, df = 1786, *p*-value = 0.045; Pearson’s correlation test), we adapted the weight of response variables to the associated precision of positions (i.e. inverse of the imprecision squared [35]) by adding a precision weight term in GLMMs.

Lights were oriented toward the ground and the armature parallel to the forest edge, which results in a heterogeneous distribution of light in horizontal and vertical planes (Fig. 1). Thus, in order to assess the dependence of light effects on bats to their spatial position around a streetlight, we built one model per species group for (i) all positions around the light, for (ii) positions under the light (i.e. *z* < 4 m), for (iii) positions above the light (*z* > 4 m), for (iv) positions at the backside of the light (i.e. *x* > 4 m) and for (v) positions in front of the light (i.e. *x* < 4 m) (Fig. 1). All GLMMs exhibited much smaller Akaike Information Criteria (AIC) than null models. We assessed for each model the goodness of fit by computing the percentage of variance explained by models using the *r2* function (R package *sjstats*). We checked residual plots of models using the R package *DHARMa.* All analyses were performed using a significance threshold of 5% in the R statistical software [36].

## Results

### 3D acoustic localization

Bat calls within the 25,195 five-second files recorded allowed the assessment of a total of 28,646 (3D) positions, with an imprecision of less than one meter. Of all positions, 91.3% were assigned to the *Pipistrellus* group, 6.2% to the *Eptesicus/Nyctalus* group and 2.4% to the *Myotis/Plecotus* group (Additional file 1: Table S2). The number of locations was higher at the white-light poles, followed by red and then dark control poles for *Eptesicus/Nyctalus* and *Pipistrellus* groups, but higher around red-light poles followed by white and then dark control poles for *Myotis/Plecotus* group (Additional file 1: Table S2). Overall, the cumulative imprecision for each location varied between 0.10 and 0.39 m on average, and was dependent on species group, but similar between the three light treatments although slightly lower for all groups in red sites (Additional file 1: Table S2). More than 70% of positions had a cumulative imprecision lower than 0.2 m (Fig. S1).

### Effect of spectrum on the flight behaviour

**Fig. 2:**
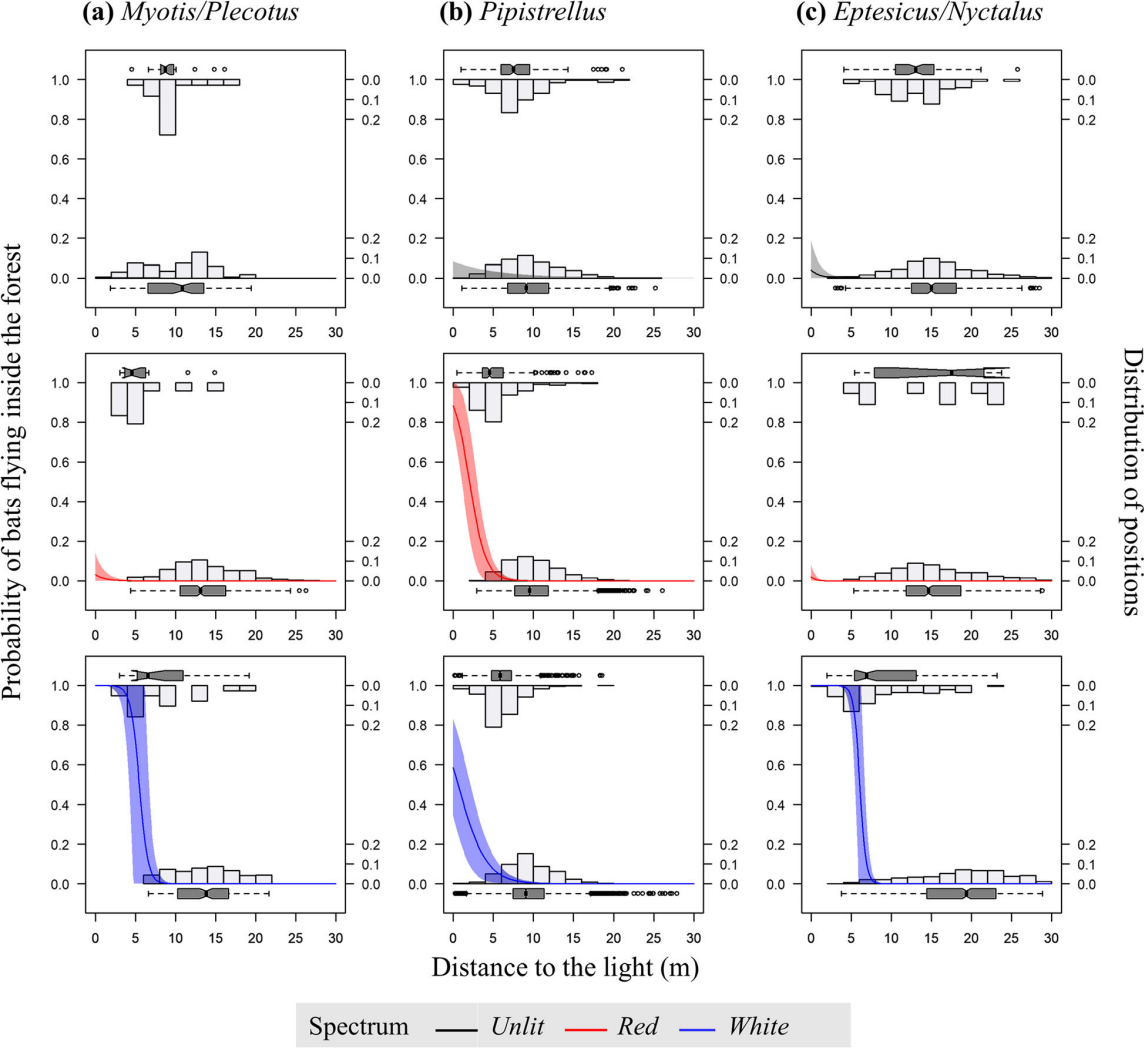
Predicted curves of the probability of bats flying inside the forest (left y axis) in relation with the distance to the light for unlit control sites (with dummy light posts), red lit sites and white lit sites. Histograms and boxplots represent the frequency distribution of bat positions (right y axis) for positions located in open area (at the bottom) and inside the forest (at the top) in relation with the distance to the light

Overall, the average probability of bats flying inside the forest (PFIF) was significantly higher near red and white light posts compared to dark control poles for *Pipistrellus* group, while only significantly higher near white light posts for *Myotis/Plecotus* and *Eptesicus/Nyctalus* groups (Table 1). All bat groups were found to have a greater PFIF when getting closer to the light. The increase in PFIF when getting closer to the light was stronger for red and white light posts compared to dark control poles for *Pipistrellus* and *Eptesicus/Nyctalus* groups, and only stronger for white light posts for the *Myotis/Plecotus* group. The increase in PFIF when getting closer to the light was even stronger for red compared to white light posts for the *Pipistrellus* group, and even stronger for white compared to red light posts for *Eptesicus/Nyctalus* (Table 1; Fig. 2). Irrespective of these differences, white lighting increased the Flight Path Switch Distance (FPSD) for *Eptesicus/Nyctalus* group (i.e. 6.1 m), red lighting generated a greater FPSD for *Pipistrellus* group (i.e. 2.0 m), and white light generated a FPSD of 5.5 m for *Myotis/Plecotus* group (Additional file 1: Table S3; Fig. 2).

**Table 1:** Estimates, standard errors and *p*-values of the effect of the distance to the light, the spectrum and the mutual interaction on the probability of bats flying inside the forest when unlit control (A) and white spectrum (B) were used as intercept (***p < .001, **p < .01, *p < .05). Results are presented for all positions, and positions above, under, behind and in front of the light (see Fig. 1 for placement definitions), and derived from generalized linear mixed models

		*Eptesicus/Nyctalus*	*Myotis/Plecotus*	*Pipistrellus*
***All positions***		*N*=1788	*N*=692	*N*=26,166
Dist. to light		−0.950 ± 0.020 ***	−1.864 ± 0.736 *	−0.188 ± 0.036 ***
Spectrum	(A) Unlit vs. Red	−0.679 ± 4.278	3.056 ± 4.055	5.185 ± 1.570 ***
	(A) Unlit vs. White	18.890 ± 4.004 ***	16.736 ± 7.994 *	3.522 ± 1.491*
	(B) White vs. Red	−19.569 ± 3.502 ***	−13.680 ± 8.379	1.663 ± 1.509
Dist. to light: Spectrum	(A) Unlit vs. Red	−1.013 ± 0.044 ***	−0.508 ± 0.376	−0.802 ± 0.067 ***
	(A) Unlit vs. White	−1.639 ± 0.435 ***	−1.537 ± 0.742 *	−0.385 ± 0.040 ***
	(B) White vs. Red	0.625 ± 0.055 ***	1.029 ± 0.790	−0.417 ± 0.059 ***
***Vertical location: above light***		*N*=1708	*N*=465	*N*=19,438
Dist. to light		−0.951 ± 0.020 ***	−1.715 ± 794 *	−0.207 ± 0.038 ***
Spectrum	(A) Unlit vs. Red	37.339 ± 8.043 ***	−4.277 ± 6.453	2.587 ± 1.690
	(A) Unlit vs. White	20.557 ± 3.787 ***	7.801 ± 8.163	2.721 ± 1.596 .
	(B) White vs. Red	16.784 ± 8.092 *	−12.078 ± 8.351	−0.134 ± 1.623
Dist. to light: Spectrum	(A) Unlit vs. Red	−6.282 ± 0.187 ***	0.783 ± 0.914	−0.477 ± 0.074 ***
	(A) Unlit vs. White	−1.487 ± 0.044 ***	−0.507 ± 1.059	−0.369 ± 0.043 ***
	(B) White vs. Red	−4.795 ± 0.190 ***	1.290 ± 0.856	−0.108 ± 0.066
***Vertical location: under light***		*N*=80	*N*=227	*N*=6728
Dist. to light		/	/	−0.567 ± 0.043 ***
Spectrum	(A) Unlit vs. Red	/	/	10.751 ± 2.080 ***
	(A) Unlit vs. White	/	/	5.340 ± 1.944 **
	(B) White vs. Red	/	/	5.411 ± 1.480 ***
Dist. to light: Spectrum	(A) Unlit vs. Red	/	/	−1.624 ± 0.197 ***
	(A) Unlit vs. White	/	/	−0.581 ± 0.162 ***
	(B) White vs. Red	/	/	−1.044 ± 0.128 ***
***Horizontal location: backside***		*N*=895	*N*=326	*N*=6433
Dist. to light		−0.520 ± 0.380	/	−0.347 ± 0.548
Spectrum	(A) Unlit vs. Red	−47.725 ± 29.775	/	−10.136 ± 7.872
	(A) Unlit vs. White	−13.521 ± 7.152 .	/	−5.289 ± 7.145
	(B) White vs. Red	−34.205 ± 28.608	/	−4.847 ± 3.978
Dist. to light: Spectrum	(A) Unlit vs. Red	1.977 ± 1.134 .	/	0.715 ± 0.588
	(A) Unlit vs. White	0.676 ± 0.408 .	/	0.429 ± 0.559
	(B) White vs. Red	1.302 ± 1.026	/	0.286 ± 0.240
***Horizontal location: front side***		*N*=893	*N*=366	N=19,733
Dist. to light		−0.748 ± 157 ***	0.959 ± 0.602	−0.555 ± 0.021 ***
Spectrum	(A) Unlit vs. Red	1.082 ± 3.269	18.784 ± 9.528 *	7.343 ± 1.893***
	(A) Unlit vs. White	6.229 ± 2.215 **	45.356 ± 20.498 *	4.534 ± 1.818 *
	(B) White vs. Red	−5.147 ± 3.384	−26.572 ± 19.127	2.809 ± 1.779
Dist. to light: Spectrum	(A) Unlit vs. Red	−0.538 ± 0.326 .	− 2.587 ± 1.036 *	−1.070 ± 0.079 ***
	(A) Unlit vs. White	−0.558 ± 0.170 **	−5.466 ± 2.590 *	−0.468 ± 0.047 ***
	(B) White vs. Red	0.020 ± 0.338	2.879 ± 2.555	−0.602 ± 0.070 ***

At three to five metres from the light, the PFIF for *Myotis/Plecotus* and *Eptesicus/Nyctalus* groups even reached 100% for white lighting treatment, while the usual PFIF at such distance in unlit sites was under 1% (Fig. 2). Similarly, the PFIF for *Pipistrellus* group reached more than 85 and 50% at one meter from red and white lights respectively, while close to 0% in unlit conditions whatever the distance (Fig. 2).

### Variation of responses according to location around lights

**Fig. 3:**
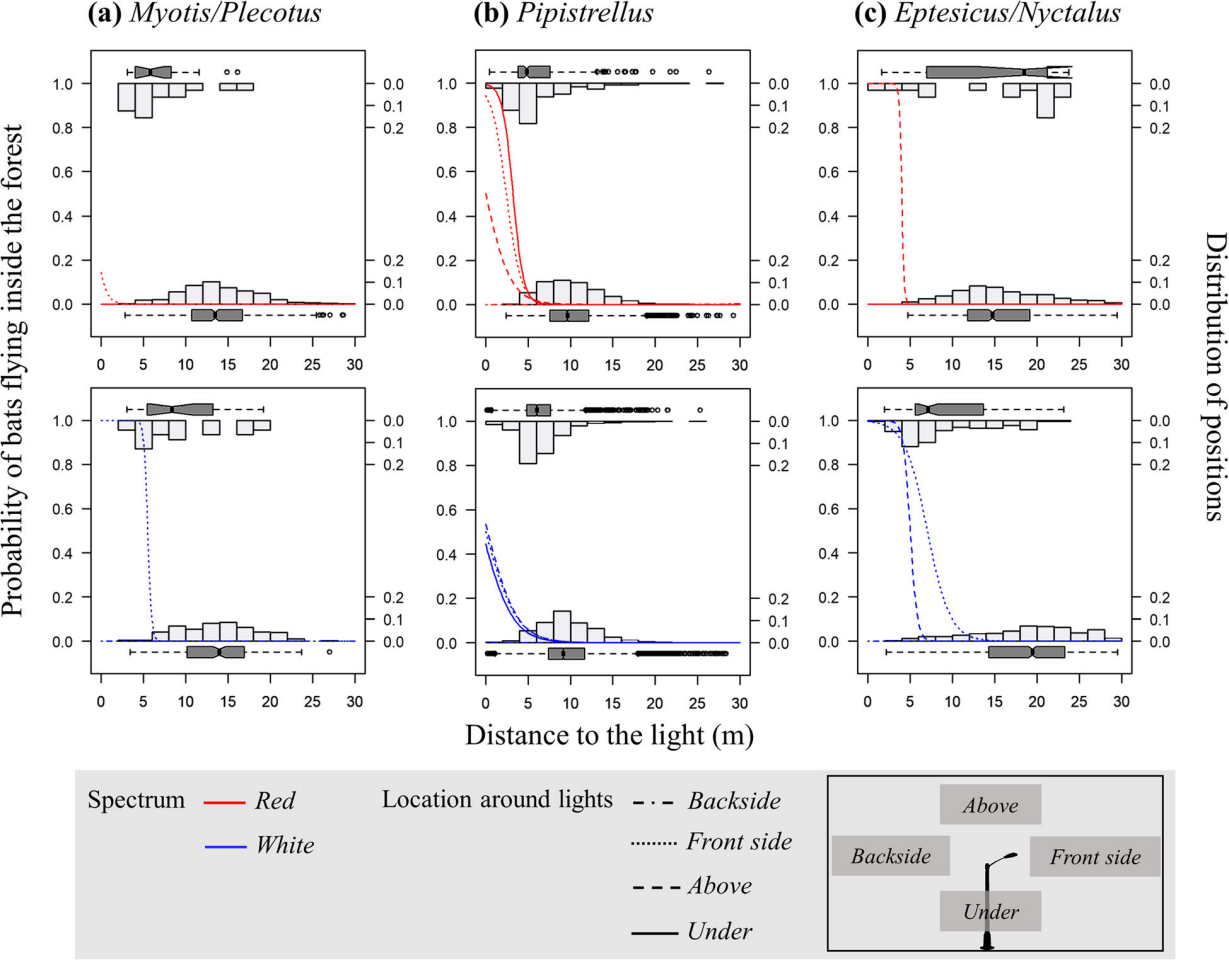
Predicted curves of the probability of bats flying inside the forest (left y axis) in relation with the distance to the light for positions at the backside of streetlight, at the front side of streetlight, above and under streetlight for red and white lit sites. Histograms and boxplots represent the frequency distribution of bat positions (right y axis) for positions located in open area (at the bottom) and inside the forest (at the top) in relation with the distance to the light

All species groups increasingly flew inside the forest when getting closer to the light. For both spectra, this effect was only present at the front side of the light, except for *Eptesicus/Nyctalus* group around red lights (Table 1; Fig. 3). For this group, the response was furthermore limited for bats flying above light posts of both spectra. For the *Pipistrellus* group, this response was unrelated to flight height (Table 1; Fig. 3).

Concerning the distance dependency, Flight Path Switch Distance (FPSD) was greater for *Eptesicus/Nyctalus* flying above and at the front side of white compared to red light poles (5.1 m and 7.0 m versus 4.0 m and 0.2 m, respectively), and was greater for *Myotis/Plecotus* flying at the front side of white light poles (6.7 m versus no prediction possibility due to insufficient PFIF, respectively) (Additional file 1: Table S3; Fig. 3). The FPSD was also higher under and at the front side of red-light poles compared to white light poles for *Pipistrellus* group (3.2 m and 2.4 m versus no prediction possibility due to insufficient PFIF, respectively) (Additional file 1: Table S3; Fig. 3).

Finally, fixed effects of models overall almost always explained a large part of the variance (0.11–0.90 r squared; Additional file 1: Table S3).

## Discussion

We show that artificial light located at forest edges significantly increases the Probability of Flying Inside the Forest (PFIF) for open-, edge- and narrow-space foragers (*Eptesicus/Nyctalus*, *Pipistrellus* and *Myotis/Plecotus* groups, respectively) regardless of light spectrum.

For open- and edge-space foraging bats that take advantage of around light accumulated insects, the presence of cluttered habitat (i.e. forest in our case) could further facilitate foraging around streetlights by providing shelter against predators. This result is also consistent with the antagonist effects of ALAN at different spatial scales for open- and edge-space foragers. At the streetlight scale these groups can appear light-opportunistic [13, 14, 21, 23, 24], however, at a larger scale these species are negatively impacted by ALAN [17–19]. The observation that light-opportunistic open- and edge-space foragers seek refuge in cluttered environment near light sources may explain this negative impact, especially in areas with little vegetation around light.

Comparable behavioural changes were expected for *Myotis/Plectus* species as they are adapted to fly in cluttered environments and are known to be light-averse [14, 23, 30]. Both red and white light increased the PFIF for *Myotis/Plecotus* group compared to dark control sites. Although the effect of red lights was much less important compared to white lights, this finding is important as red light has been reported to have limited to absent effects on the activity of these species [21, 23]. Our results thus suggest that red light may actually not be entirely effective in avoiding behavioural changes of narrow-space foragers, and even less for open- and edge-space foragers.

Overall, Flight Path Switch Distances (FPSD) were mostly longer around white lights, which is likely due to the fact that bats may perceive white light as more intense compared to red light due their spectral sensitivity [37, 38]. Such differences in FPSD could also be linked with differences in light intensity at equal distance, higher for white than red lamps we studied (4.83 more lux in average in a 5 m radius around red lights; see Additional file 1: Appendix S1 for graphical representation of light intensity in relation to the distance to white and red lights), which is known to be one of light parameters driving impacts on bats [39]. When considering the vertical location of bat positions, we found that compared to white light poles, the FPSD was higher for individuals located under (i.e. for *Pipistrellus* group) and above (i.e. for *Pipistrellus* and *Eptesicus/Nyctalus* groups) red light poles. This is likely directly related to the distribution of the light around the streetlights (Additional file 1: Appendix S1), and aligns with the distance relation – and hence intensity dependence – of activity reported by [24]. However, it should be noted that *Eptesicus/Nyctalus* group mainly flew above lights (Additional file 1: Table S2) which likely explain the absence of response under lights.

We also found changes in bat behaviour in front of light posts for all groups but not at the backside. Thus, the directionality of the light post matters and can be used to reduce the adverse impacts of artificial lighting on bats. In our study, we had a sharper cut-off in light at the backside of the light posts, and hence the effects there disappeared at shorter distances. Individuals could forage at the backside of streetlights where the predation risk is low, and hence not seek refuge inside forest when getting closer to the light. Further investigations are needed to understand mechanisms involved. Concerning the overall higher effect of red light compared to white light on *Pipistrellus* group, further studies would be needed to understand why, and if they possibly turn back when getting closer to light instead of seeking refuge inside the forest. However, open-space foragers do not show the same pattern and react similarly to red and white light. We could hypothesise that their higher flight height allows for flying in or above the canopy (i.e. in a potentially more open space than for *Pipistrellus* group) while increasing their flight speed in response to light, which could explain their different response than edge-space foragers. Further investigations are also needed to address these aspects.

Finally, depending on bat location around streetlights, Flight Path Switch Distances (FPSD) in front of streetlights overall started from 7 m and 4 m for white and red lights, respectively. These distances of impact correspond to light intensities around 6 lux for both white and red lights. However, we defined FPSD as the distance at which bats on average flew as much inside as outside the forest, but impacts likely start before this arbitrarily chosen threshold. When we look at the beginning of behavioural perturbation, i.e. when the PFIF previously close to zero increases towards positive PFIFs, corresponding FPSD would be around 15 m and 9 m for white and red lights, respectively. Such distances correspond to light intensities around one lux for both white and red lights. These thresholds seem to be consistent with a study which looked at thresholds in light intensity affecting bat activity [24].

However, it is important to be cautious about the definition of safety thresholds for bats and further studies should confirm these results by testing wider distance and intensity ranges around streetlights, by sampling all spectra simultaneously, and by studying more sites and nights per spectrum. We were not able to measure forest height, thus further studies could accurately account for bat position in relation to forest canopy as open space foragers such as *Nyctalus* spp. can fly above (average flight height 9±4 m; Additional file 1: Fig. S3). However, considerably lower flight heights recorded for *Pipistrellus* (5.8±2.8 m) and *Myotis/Plecotus* groups (5.2±2.5 m), and light effects generalized to under and above light positions for *Pipistrellus* group support these findings (Additional file 1: Fig. S3).

## Conclusion

Our study demonstrates that spectrum type, intensity and directionality of streetlights has an effect on the flight behaviour of all bats, including light-opportunistic species, highlighting the need to consider simultaneously all these characteristics when studying ALAN impact on bats. In contrast to the absence of changes in bat activity in response to red light reported earlier, we here show that bats can have a comparable change in flight behaviour in response to red and white light. This finding first shows that bats actively seek refuge in cluttered environment when getting closer to light sources. This is a clear indication that bats make use of landscape structures when dealing with light, and shows the potential of vegetation in mitigating negative impacts of artificial light at night, but calls for preserving darkness as the most efficient way.

## Supplementary Information

**Additional file 1.**

## Data Availability

R scripts and data used for analyses are available in the Zenodo repository, 10.5281/zenodo.4036279.
