# Supplementary material for: Bats seek refuge in cluttered environment when exposed to white and red lights at night
Source: Mov Ecol. 2021 Jan 22;9:3. doi: 10.1186/s40462-020-00238-2 (PMC7821510; doi:10.1186/s40462-020-00238-2)

**Supplementary material to :**

**Bats seek refuge in cluttered environment when exposed to white and red lights at night**

Kévin Barré, Christian Kerbiriou, Ros-Kiri Ing, Yves Bas, Clémentine Azam, Isabelle Le Viol, Kamiel Spoelstra

**Figure S1. Histogram of the proportion of 3D positions in relation to the imprecision computed on the three dimensions for selected data with an imprecision < 1 meter.**


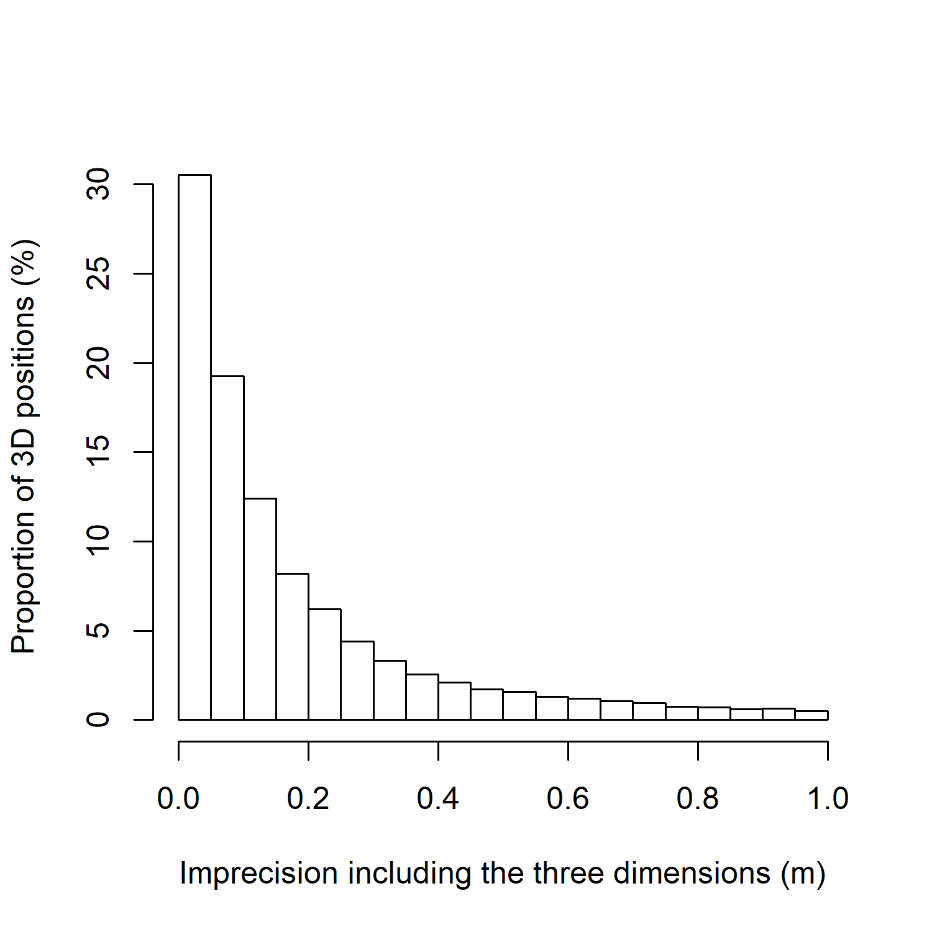


**Table S1.** Weather conditions at the beginning of each sampling session.

| Sampling date | Spectrum | Temperature (°C) | Humidity (%) | Wind speed (km/h) | Rain (mm) |
| --- | --- | --- | --- | --- | --- |
| 10/07/2018 | Red | 13.6 | 75 | 4 | 0 |
| 11/07/2018 | Control | 16.2 | 82 | 7 | 0 |
| 12/07/2018 | White | 14.9 | 88 | 4 | 0 |
| 13/07/2018 | Control | 15.2 | 82 | 4 | 0 |
| 14/07/2018 | Red | 15.3 | 75 | 4 | 0 |
| 16/07/2018 | Control | 15.7 | 83 | 0 | 0 |
| 17/07/2018 | White | 14.6 | 83 | 7 | 0 |
| 18/07/2018 | Red | 17.9 | 65 | 4 | 0 |
| 19/07/2018 | Control | 16.1 | 78 | 4 | 0 |
| 20/07/2018 | White | 19.3 | 62 | 11 | 0 |
| 21/07/2018 | Red | 19.0 | 77 | 7 | 0 |
| 22/07/2018 | White | 16.0 | 84 | 0 | 0 |

**Table S2**. Number of positions around the streetlights with the mean and standard deviation of their imprecision (m) for the three dimensions.

|  | Control | |  | Red spectrum | |  | White spectrum | |  | Total | |
| --- | --- | --- | --- | --- | --- | --- | --- | --- | --- | --- | --- |
|  | Positions | Imprecision |  | Positions | Imprecision |  | Positions | Imprecision |  | Positions | Imprecision |
| ***All positions*** |  |  |  |  |  |  |  |  |  |  |  |
| *Eptesicus/Nyctalus* | 447 | 0.386 ± 0.252 |  | 505 | 0.263 ± 0.239 |  | 836 | 0.367 ± 0.243 |  | 1788 | 0.345 ± 0.249 |
| *Myotis/Plecotus* | 103 | 0.288 ± 0.255 |  | 412 | 0.151 ± 0.166 |  | 177 | 0.213 ± 0.200 |  | 692 | 0.191 ± 0.199 |
| *Pipistrellus* | 2318 | 0.183 ± 0.201 |  | 6894 | 0.100 ± 0.137 |  | 16950 | 0.195 ± 0.208 |  | 26166 | 0.169 ± 0.196 |
| ***Vertical location: above light*** | |  |  |  |  |  |  |  |  |  |  |
| *Eptesicus/Nyctalus* | 433 | 0.394 ± 0.252 |  | 465 | 0.268 ± 0.242 |  | 810 | 0.376 ± 0.242 |  | 1708 | 0.353 ± 0.249 |
| *Myotis/Plecotus* | 55 | 0.177 ± 0.174 |  | 272 | 0.156 ± 0.160 |  | 138 | 0.177 ± 0.174 |  | 465 | 0.193 ± 0.192 |
| *Pipistrellus* | 1787 | 0.214 ± 0.212 |  | 3827 | 0.124 ± 0.152 |  | 13824 | 0.210 ± 0.212 |  | 19438 | 0.194 ± 0.204 |
| ***Vertical location: under light*** | |  |  |  |  |  |  |  |  |  |  |
| *Eptesicus/Nyctalus* | 14 | 0.260 ± 0.231 |  | 40 | 0.222 ± 0.201 |  | 26 | 0.200 ± 0.218 |  | 80 | 0.222 ± 0.214 |
| *Myotis/Plecotus* | 48 | 0.191 ± 0.231 |  | 140 | 0.141 ± 0.177 |  | 39 | 0.328 ± 0.233 |  | 227 | 0.187 ± 0.213 |
| *Pipistrellus* | 531 | 0.112 ± 0.153 |  | 3067 | 0.070 ± 0.108 |  | 3130 | 0.129 ± 0.176 |  | 6728 | 0.102 ± 0.151 |
| ***Horizontal location: backside*** | | |  |  |  |  |  |  |  |  |  |
| *Eptesicus/Nyctalus* | 76 | 0.349 ± 0.221 |  | 235 | 0.325 ± 0.243 |  | 584 | 0.429 ± 0.228 |  | 895 | 0.370 ± 0.236 |
| *Myotis/Plecotus* | 26 | 0.391 ± 0.243 |  | 216 | 0.209 ± 0.182 |  | 84 | 0.260 ± 0.206 |  | 326 | 0.240 ± 0.202 |
| *Pipistrellus* | 489 | 0.292 ± 0.227 |  | 1492 | 0.166 ± 0.165 |  | 4452 | 0.318 ± 0.253 |  | 6433 | 0.280 ± 0.242 |
| ***Horizontal location: front side*** | | |  |  |  |  |  |  |  |  |  |
| *Eptesicus/Nyctalus* | 371 | 0.394 ± 0.258 |  | 270 | 0.208 ± 0.222 |  | 252 | 0.230 ± 0.220 |  | 893 | 0.291 ± 0.252 |
| *Myotis/Plecotus* | 77 | 0.250 ± 0.250 |  | 196 | 0.083 ± 0.111 |  | 93 | 0.169 ± 0.185 |  | 366 | 0.146 ± 0.186 |
| *Pipistrellus* | 1829 | 0.154 ± 0.184 |  | 5402 | 0.036 ± 0.119 |  | 12502 | 0.147 ± 0.165 |  | 19733 | 0.130 ± 0.159 |

**Appendix S1. Relationship between the light intensity and the distance to the lamp**

In this study we tested the influence of the distance of the 3D positions of bats to the lamp as a proxy of the lighting intensity at a given spatial bat location. In order to facilitate the extrapolation of our results to light intensity, we modelled the relationship between the lighting intensity and the distance to the light (see Figure below). For that we measured the light intensity at ground level using a highly sensitive lux meter (LMT B360, LMT Lichtmesstechnik, Berlin, Germany) along three 30 m transects, with an interval of one m for the first 16 m, followed by an interval of two m thereafter. Light was measured at two transects along the forest edge, one at the backside (dotted lines) and one in front of the light (solid lines) (Fig. 1), and one perpendicular to the edge (dashed lines), each starting from the streetlight.


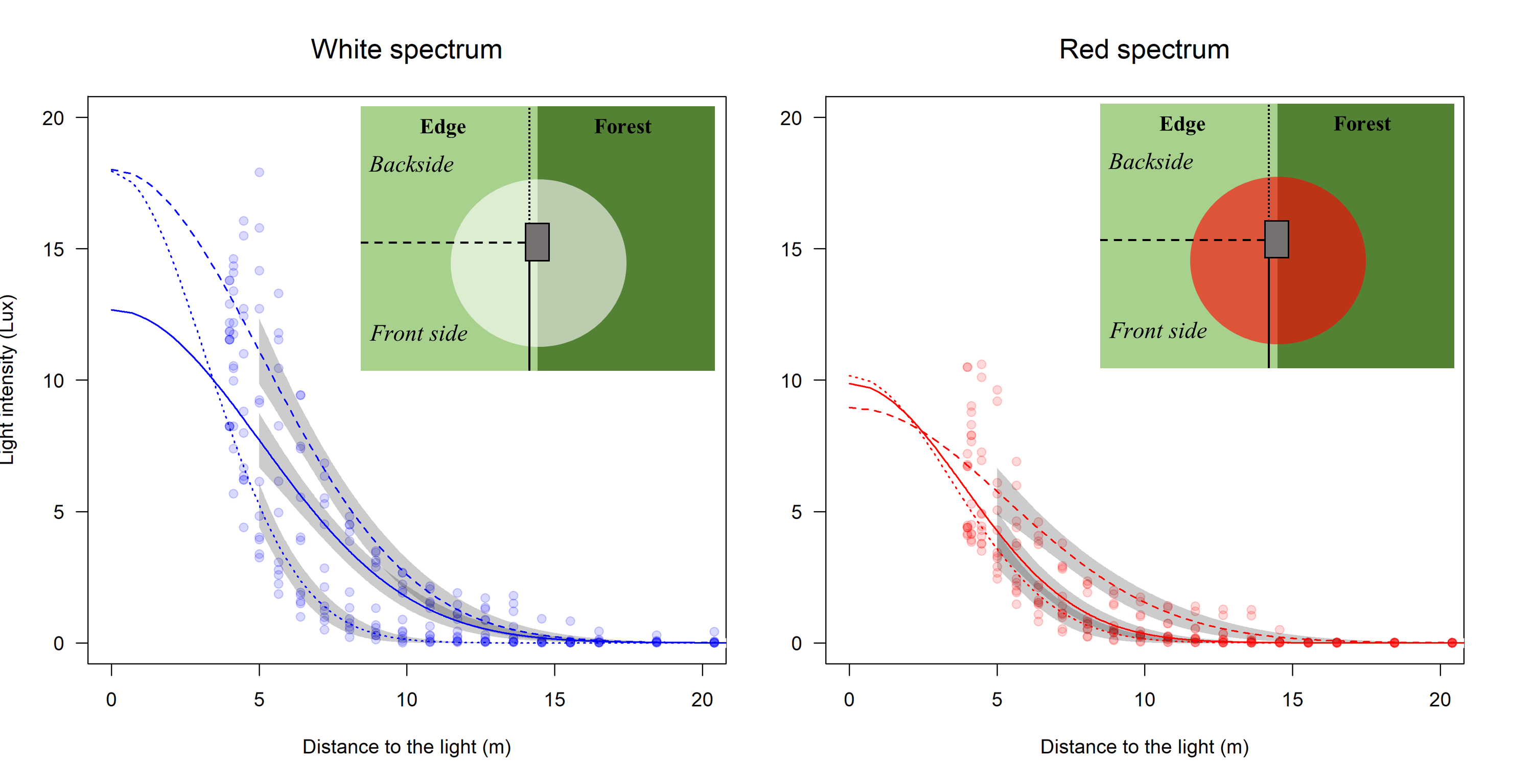


We modelled light intensity as a function of the square of the distance to the light using generalized linear models associated with a Poisson error distribution (R package *stats*).

The light intensity behind the streetlight decreased more quickly with distance than perpendicular and front transects of light measurements for white light. Although red lights showed the same pattern, the differences between light levels behind and in front of the streetlight are less obvious (Fig. 4).

**Appendix S2. Precisions about sound recording, triggering of bat echolocation call recording and time of call arrival differences (TOAD)**

Signals from microphones are pre-amplified by a custom-made electronic device of 100 kHz bandwidth and 40 dB gain. An analog to digital converter (Agilent U2542A, Santa Clara, California) was used to digitize analogous signals (in 16 bits format). A portable rugged computer was used for real time computation of 3D positions of bat calls. The protocol for the calculation of 3D positions is detailed in Ing et al. (2016). The recording of bat calls is triggered when the central microphone records a signal power exceeding a threshold within the 20-100 kHz frequency band, over a duration of 3.2ms. When the power averaged over this interval is eight times higher than that of the noise within the same frequency band averaged over 10 seconds the system records the signal of all four microphones. At each trigger event, the signals of the call are recorded for a duration of 51.2ms.

Ing, R.K., Colombo, R., Gembu, G.-C., Bas, Y., Julien, J.-F., Gager, Y., Hassanin, A., 2016. Echolocation Calls and Flight Behaviour of the Elusive Pied Butterfly Bat ( Glauconycteris superba ), and New Data on Its Morphology and Ecology. Acta Chiropterologica 18, 477–488. <https://doi.org/10.3161/15081109ACC2016.18.2.014>

**Appendix S3. Trajectory reconstruction**

Starting from reconstructed positions in 3D, we wrote a R script allowing to reconstruct most likely trajectories. The script is based on following criteria:

- We grouped positions in blocks (step 1), i.e. a new block was defined when two positions were more than 2 seconds apart.

In each block we checked (step 2):

- the position is in the same file as the previous (i.e. same night of recording)
- the time difference is above 2 seconds
- this position's identifier is the previous position's identifiers + 1 based on time of recording

Then we computed speeds of flight as follows (V, equation 1):


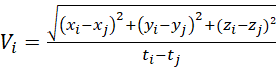
 (Eq. 1)

where *x*, *y* and *z* represent distances to the microphone array for each of the three-dimension axis, and *t* is the time of call arrival to the microphone array of a given position *i* and its previous position *j*.

We then discarded positions for which (step 3):

- the energy peak was different from the median of the block's energy peaks +/- 5 kH
- the speed of the line to add is higher than the sum of the speeds of the two following positions
- the speed is higher than 30 m/s

Finally, we re-ran these 3 steps on discarded positions to attempt to assign them to other trajectories (e.g. trajectories of other species).

Because speeds were potentially computed before a removing of previous positions for a given position, after trajectory reconstruction steps we re-run speed computing (see Equation 1) for each position included in final trajectories.

**Figure S2. Histogram of the number of positions inside trajectories.**


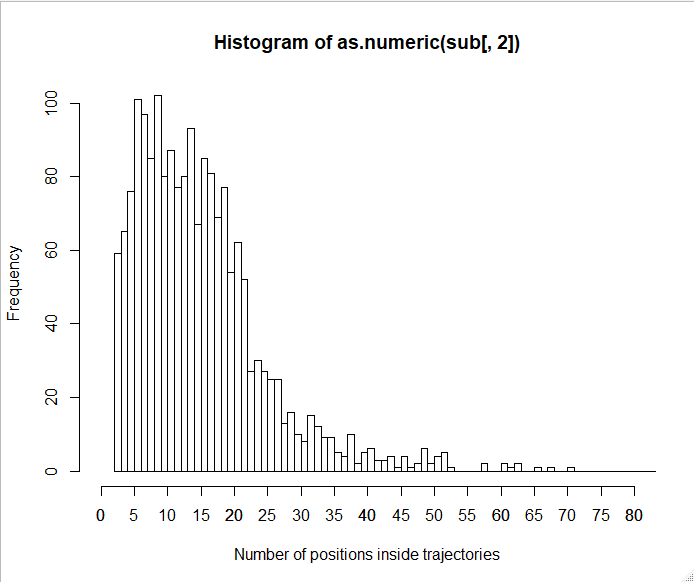


**Table S3. Model formulas, Akaike Information Criterion differences with null models (delta AIC), predicted flight path switch distances and explained variance by fixed effects (Marginal R2), by spectrum and by distance to the light (adjusted R2) for all positions, and positions above, under, behind and in front of the light.**

| Species group | Model formula | delta AIC (*vs.* null model) | Flight path switch distance | |  | Explained variance | | | |
| --- | --- | --- | --- | --- | --- | --- | --- | --- | --- |
| ***All positions*** |  |  | Red | White |  | Marginal R2 | R2 adjsusted to spectrum | R2 adjusted to dist. to light |  |
| *Eptesicus/Nyctalus* | Spectrum * Dist. to light + (1|Site) + (1|Trajectory) | -24164 | / | 6.1 m |  | 0.895 | 0.551 | 0.179 |  |
| *Myotis/Plecotus* | Spectrum * Dist. to light + (1|Site) + (1|Trajectory) | -55 | / | 5.5 m |  | 0.895 | 0.274 | 0.756 |  |
| *Pipistrellus* | Spectrum * Dist. to light + (1|Site) + (1|Trajectory) | -4185300 | 2.0 m | 0.6 m |  | 0.412 | 0.036 | 0.262 |  |
| ***Vertical location: above light*** | |  |  |  |  |  |  |  |  |
| *Eptesicus/Nyctalus* | Spectrum * Dist. to light + (1|Site) + (1|Trajectory) | -26186 | 4.0 m | 5.1 m |  | 0.536 | 0.523 | 0.207 |  |
| *Myotis/Plecotus* | Spectrum * Dist. to light + (1|Site) + (1|Trajectory) | -36 | / | / |  | 0.853 | 0.240 | 0.847 |  |
| *Pipistrellus* | Spectrum * Dist. to light + (1|Site) + (1|Trajectory) | -1584 | / | 0.2 m |  | 0.302 | 0.022 | 0.228 |  |
| ***Vertical location: under light*** | |  |  |  |  |  |  |  |  |
| *Eptesicus/Nyctalus* | Spectrum * Dist. to light + (1|Site) + (1|Trajectory) | -242 | / | / |  | 0.600 | 0.957 | 0.030 |  |
| *Myotis/Plecotus* | Spectrum * Dist. to light + (1|Site) + (1|Trajectory) | / | / | / |  | / | / | / |  |
| *Pipistrellus* | Spectrum * Dist. to light + (1|Site) + (1|Trajectory) | -590 | 3.2 m | / |  | 0.668 | 0.012 | 0.412 |  |
| ***Horizontal location: backside*** | |  |  |  |  |  |  |  |  |
| *Eptesicus/Nyctalus* | Spectrum * Dist. to light + (1|Site) + (1|Trajectory) | 1 | / | / |  | 0.072 | 0.072 | 0.039 |  |
| *Myotis/Plecotus* | Spectrum * Dist. to light + (1|Site) + (1|Trajectory) | / | / | / |  | / | / | / |  |
| *Pipistrellus* | Spectrum * Dist. to light + (1|Site) + (1|Trajectory) | 5 | / | / |  | 0.164 | 0.021 | 0.035 |  |
| ***Horizontal location: front side*** | |  |  |  |  |  |  |  |  |
| *Eptesicus/Nyctalus* | Spectrum * Dist. to light + (1|Site) + (1|Trajectory) | -67 | 0.2 m | 7.0 m |  | 0.825 | 0.217 | 0.122 |  |
| *Myotis/Plecotus* | Spectrum * Dist. to light + (1|Site) + (1|Trajectory) | -58 | / | 6.7 m |  | 0.110 | 0.271 | 0.635 |  |
| *Pipistrellus* | Spectrum * Dist. to light + (1|Site) + (1|Trajectory) | -1482 | 2.4 m | / |  | 0.360 | 0.043 | 0.160 |  |

**Figure S3. Histograms of light heights for Pipistrellus group (A), Myotis/Plecotus group (B) and Eptesicus/Nyctalus group (C).**

**(A)**

**(B)**

**(C)**


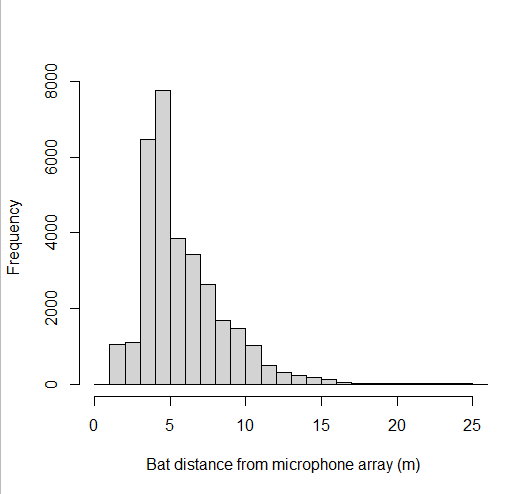

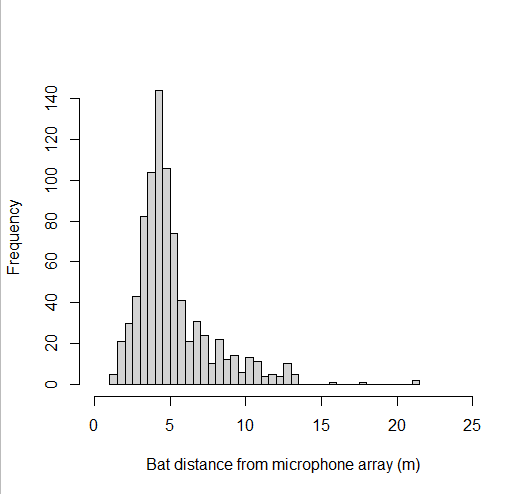

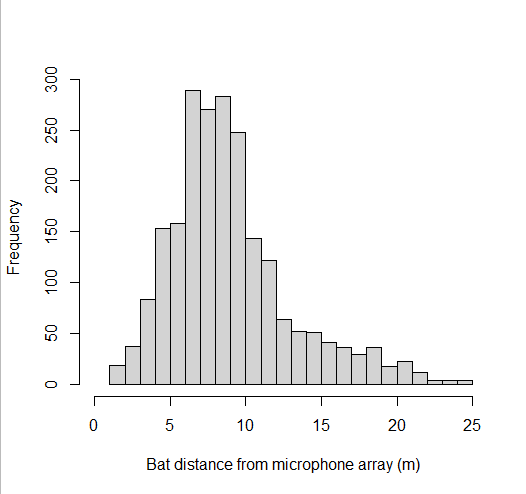

Supplement: Supplementary file 1 — Additional file 1. [file 40462_2020_238_MOESM1_ESM.doc]
